# Synthesis of hydroxy benzoin/benzil analogs and investigation of their antioxidant, antimicrobial, enzyme inhibition, and cytotoxic activities

**DOI:** 10.3906/kim-2012-25

**Published:** 2021-06-30

**Authors:** Nurettin YAYLI, Gözde KILIÇ, Gonca ÇELİK, Nuran KAHRİMAN, Şeyda KANBOLAT, Arif BOZDEVECİ, Şengül ALPAY KARAOĞLU, Rezzan ALİYAZICIOĞLU, Hasan Erdinç SELLİTEPE, İnci Selin DOĞAN, Ali AYDIN

**Affiliations:** 1 Department of Pharmacognosy, Faculty of Pharmacy, Karadeniz Technical University, Trabzon Turkey; 2 Department of Chemistry, Faculty of Science, Karadeniz Technical University, Trabzon Turkey; 3 Department of Biochemistry, Faculty of Pharmacy, Karadeniz Technical University, Trabzon Turkey; 4 Department of Biology, Faculty of Arts and Science, Recep Tayyip Erdoğan University, Rize Turkey; 5 Department of Pharmaceutical Chemistry, Faculty of Pharmacy, Karadeniz Technical University, Trabzon Turkey; 6 Department of Medical Biology, Faculty of Medicine, Yozgat Bozok University, Yozgat Turkey

**Keywords:** Hydroxy benzoin/benzil, benzoin/benzil-O-β-D-glucoside, antioxidant, antimicrobial, enzyme inhibition, cytotoxic activity

## Abstract

In this study, hydroxy benzoin ( **1-7** ), benzil ( **8-14** ), and benzoin/benzil-O-β-D-glucosides ( **15-25** ) were synthesized to investigate their biological activities. An efficient method for synthesizing hydroxy benzoin compounds ( **1** - **7** ) was prepared from four different benzaldehydes using an ultrasonic bath. Then, antioxidant (FRAP, CUPRAC, and DPPH), antimicrobial (3 Gram (-), 4/6 Gram (+), one tuberculosis and one fungus), and enzyme inhibition (acetylcholinesterase, butyrylcholine esterase, tyrosinase, α-amylase, and α- glucosidase) for the all synthesized compounds ( **1-25** ) were evaluated. And also, four most active compounds ( **4** , **12** , **18a+b** , and **25** ) from each group were evaluated to the human cervical cancer cell line (HeLa) and anticancer screening tests against the human retinal normal cell line (RPE). Compound **4** showed HeLa and RPE cancer cell activities as much as cisplatin. The synthesized compounds were characterized by spectroscopic methods (NMR, FT-IR, UV, LC-QTOF-MS) and the ACD NMR program’s help.

## 1. Introduction

It is known that drugs containing phenolic compounds are frequently used to treat diseases such as diabetes, Alzheimer’s, and cardiovascular diseases. One of the most abundant secondary metabolites in plants is phenolic compounds, such as simple phenols, phenolic acids, flavones, flavanones, and stilbenes. The main sources of phenolic compounds are fruits and vegetables, making up an important part of the human diet. According to the studies of the national health organization, due to the antioxidant effects of phenolic compounds found in herbal products, it has been revealed that rich fruit and vegetable consumption reduces the risks of diseases such as cancer, diabetes, Alzheimer’s, and cardiovascular diseases [1]. Some benzoin compounds with the phenolic structure are found in fruits and vegetables in nature.

The carbon-carbon bond formation is an important reaction in organic chemistry and studied extensively in the literature [2–5]. The benzoin condensation reaction is an important type of C-C bond formation reaction and is widely used to synthesize natural compounds and analogs. Symmetric and asymmetric benzoin derivative synthesis using different catalysts in benzoin condensation have been studied under milder reaction conditions [2–15]. However, the condensation of two different benzaldehydes may have a widely different character; only the more stable form of the isomeric mixed benzoins could be isolable in excess. When the carbonyl group is adjacent to the phenyl ring with the more electron-donating substituent, it is consistent with the reversibility of the reaction and the relative stability of the carbonyl groups in the possible products [16]. In the literature, the synthesis of mixed benzoin had been made by a variety of methods involving the generation of a “masked” acyl carbanion, which reacts with aromatic aldehydes [17], the addition of an excess of the Grignard reagent to a cyanohydrin or a protected cyanohydrin of an aromatic aldehyde [18], and reduction of unsymmetrical benzils [19]. Thus, all of the mixed benzoin synthesis involve masking or unmasking steps.

A literature search showed that various synthetic methods were reported for the benzil syntheses [20-22]. Diphenyl alkynes were oxidized efficiently to yield the corresponding benzil [23]. In another work, the selective addition of organomagnesium reagents to 2,4,6-trichlorophenyl isocyanide then following reactions leading to an efficient synthesis of benzil compounds [24]. Facile oxidation of benzylic alcohols and benzoin to give benzil compounds with various oxidation reagents had been reported [24–32].

Carbohydrates play important functional roles in numerous physiological processes, including various disease states [33–34]. Synthetic carbohydrates-based small molecule selective inhibitors are thereof being pursued as potential medicinal agents [35–38]. 

The significance of benzoin/benzil and carbohydrate-based agents caught our attention for the synthesis of benzoin/benzil-O- *β* -D-glucosides, and we decided to study their pharmacological activities. Due to the biological activities’ evaluation, we wish to report the synthesis of hydroxy benzoins ( **1-7** ) from hydroxy benzaldehydes, hydroxy benzils ( **8-14** ) from the oxidation of benzoins ( **1-7** ), and benzoin/benzil-O- *β* -D-glucosides ( **15-25** ) from the glycosylation of hydroxy benzoins/benzils ( **1-14** ). Then, their antioxidant, antimicrobial, enzyme inhibitions, and cytotoxic activity investigations were reported.

## 2. Material and methods

Solvents ( *n* -hexane, chloroform, ethyl acetate, acetone, methanol, and dimethyl sulfoxide), aldehyde compounds (benzaldehyde, 3-hydroxybenzaldehyde, 4-hydroxybenzaldehyde, and 3,5-dihydroxybenzaldehyde), and any used reagent were purchased from by Sigma-Aldrich (Sigma-Aldrich Corp., St. Louis, MO, USA), Fluka, or Merck (Merck&Co., Inc., Kenilworth, NJ, USA) unless otherwise stated. ^1^H and ^13^C NMR spectra were obtained on a Bruker 400 MHz NMR spectrometer (400 MHz for ^1^H, 100 MHz for ^13^C), using tetramethylsilane (TMS) as an internal standard. CDCl_3_, CD_3_OD, and acetone-d_6_ were used as NMR solvents. ^13^C and APT spectra were adjusted according to deutero solvent peaks. Chemical shifts were expressed in δ (ppm), and coupling constants ( *J* ) were reported in hertz (Hz). ACD NMR program was used for the interpretation of spectra. Ultrasonic bath (340 W, WiseClean, VUC-A06H) was used for the benzoin synthesis. FT-IR spectra were taken using the Perkin-Elmer 1600 (ATR) (4000–400 cm^–1^) spectrophotometer (PerkinElmer, Inc., Waltham, MA USA). Melting points were determined using the Thermo-var apparatus fitted with a microscope. Normal phase silica gel (230–400 mesh) was used in vacuum column chromatography (VLC). TLC was carried out on silica gel 60 F_254_, and the spots were visualized by ultraviolet (UV) lamp (254 nm and 366 nm) or spraying with 20% H_2_SO_4_ and heating. 

**Synthesis of hydroxy benzoins (1-7):** Hydroxy benzaldehydes (0.001 mol) in dry DMSO (10 mL) were reacted with KCN (0.001 mol) in an N_2_ environment using an ultrasonic bath (340 W, 120 min) at 70–85 ^°^C. The reactions were terminated after the TLC control. Water (30 mL) was added to the flask, extracted with ethyl acetate (3×30 mL) to give a crude mixture then compounds **1-7** were purified as a racemic mixture with repeated vacuum liquid chromatography (VLC, Silica gel 230–400 mesh) using the increasing polarity of *n* -hexane, chloroform, ethyl acetate, and methanol solvent mixtures, and the fractions were checked by TLC (Figure, Table 1). The synthesis of compounds **1** [39], **2** [16, 40–41], and **4** [42–43] had been mentioned in the literature.

**Table 1:** Experimental method for the synthesis of hydroxy benzoin compounds (1-7).

Reagents (0.01mol each)	Method	Temp.	Time	Possible benzoin productsR1PhCOCH(OH)PhR2	No	Yielda(%)
Benzaldehyde 3-HydroxybenzaldehydeKCN	US340 Watt85 oCDMSO (10 mL) N2	70–85 (oC)	60min.	R1, R2=-HR1, R2=3-OHR1=-H, R2=3-OHR1=3-OH, R2=-H	1	2408-45
Benzaldehyde 4-HydroxybenzaldehydeKCN				R1, R2=-H R1, R2=4-OHR1=-H, R2=4-OHR1=4-OH, R2=-H	2	32--48
Benzaldehyde 3,5-Dihydroxybenzaldehyde KCN				R1, R2=-HR1, R2=3,5-diOHR1=-H, R2=3,5-diOHR1=3,5-diOH, R2=-H	3	4511-40
3-HydroxybenzaldehydeKCN				R1, R2=3-OH	4	68
3-Hydroxybenzaldehyde4-HydroxybenzaldehydeKCN				R1, R2=3-OHR1, R2=4-OHR1=3-OH, R2=4-OH R1=4-OH, R2=3-OH	5	12--39
3-Hydroxybenzaldehyde3,5-Dihydroxybenzaldehyde KCN				R1, R2=3-OHR1, R2=3,5-di-OHR1/R2=3,5-diOH, R2/R1=3-OH	6a+b	171455
3,5-Dihydroxybenzaldehyde KCN				R1, R2=3,5-di-OH	7	65

aStarting aldehydes were also observed

**Figure:**
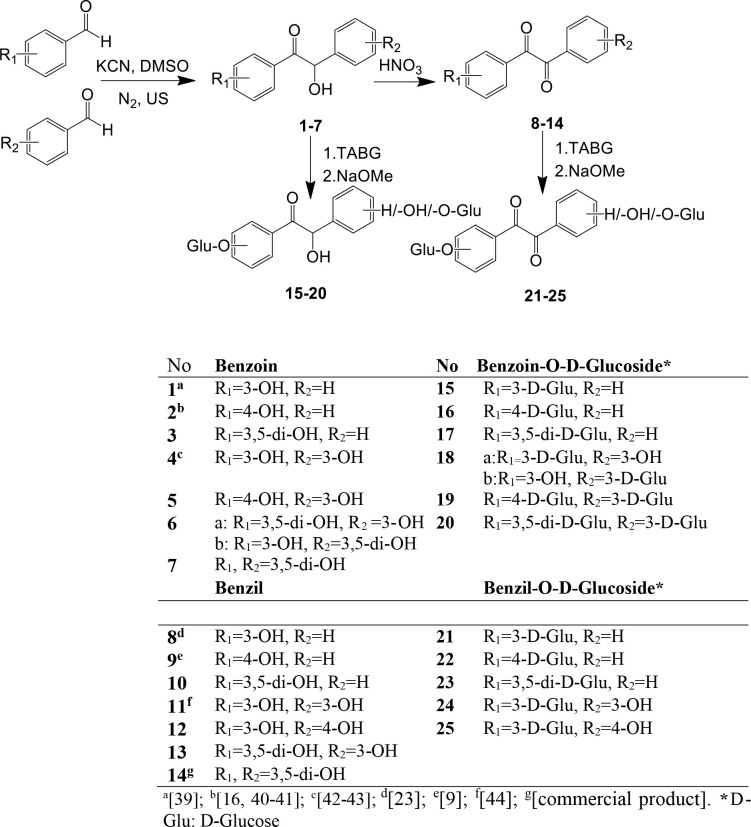
Synthesis scheme for the hydroxy benzoin, benzil, and their D-glucoside derivatives (R1 and R2: -H, -OH, or D-Glucose).

**Compound 1 (2-Hydroxy-1-(3-hydroxyphenyl)-2-phenylethanone):** Yield: 45%; R_f _= 0.5 (chloroform-ethyl acetate-acetic acid: 25:10:1); UV (MeOH) λ max nm (logɛ): 203(3,37); FT-IR (cm^–1^): 3198, 2924, 1682, 1597, 1584, 1485, 1450, 1285, 1240, 1068, 1014, 950, 787, 762, 700; ^1^H-NMR (400 MHz, CDCl_3_, d, ppm): 5.82 (s, 1H, H-2), 7.37–7.02 (m, 9H, H-2’/4’/5’/6’/2’’/3’’/4’’/5’’/6’’), 4.80 (bs, -OH); ^13^C-NMR (100 MHz, CDCl_3_, d, ppm): 198.86 (C-1), 76.16 (C-2), 130.34 (C-1’), 115.71 (C-2’), 156.74 (C-3’), 121.07 (C-4’), 134.64 (C-5’), 121.55 (C-6’), 138.60 (C-1’’), 127.79 (C-2’’), 129.13 (C-3’’), 119.28 (C-4’’), 129.13 (C-5’’), 128.67 (C-6’’). 

**Compound 2 (2-Hydroxy-1-(4-hydroxyphenyl)-2-phenylethanone)** : Yield: 48%; R_f_ = 0.48 (chloroform-ethyl acetate-acetic acid: 25:10:1); FT-IR (cm^–1^): 3371, 3040, 2920, 1661, 1584, 1514, 1455, 1388, 1260, 1065, 971, 836, 763, 701; ^1^H-NMR (400 MHz, CD_3_OD_,_ d, ppm): 6.05 (s, 1H, H-2), 7.90 (d, *J* = 8.0 Hz, 2H, H-2’/6’), 6.78 (d, *J* = 8.0 Hz, 2H, H-3’/5’), 7.43 (d, *J* = 8.0 Hz, 2H, H-2’’/6’’), 7.34 (t, *J* = 8.0 Hz, 2H, H-3’’/5’’), 7.26 (d, *J* = 8.0 Hz, 1H, H-4’’), 8.52 (bs, Ar *-* OH); 5.02 (bs, 1H, *-* OH); ^13^C-NMR (100 MHz, CD_3_OD, d, ppm): 197.34 (C-1), 75.45 (C-2), 125.90 (C-1’), 131.35 (C-2’), 114.83 (C-3’), 162.65 (C-4’), 114.83 (C-5’), 131.35 (C-6’), 139.74 (C-1’’), 127.42 (C-2’’), 128.45 (C-3’’), 127.88 (C-4’’), 128.45 (C-5’’), 127.42 (C-6’’). 

**Compound 3 (2-Hydroxy-1-(3,5-dihydroxyphenyl)-2-phenylethanone):** Yield: 40%; R_f _= 0.48 (chloroform-ethyl acetate-acetic acid: 25:10:1); brown oil; UV (MeOH) λ max nm (logɛ): 213 (2,57); FT-IR (cm^–1^): 3367, 3028, 2960, 1681, 1598, 1452, 1341, 1304, 1164, 1082, 1036, 1004, 699; ^1^H-NMR (400 MHz, CDCl_3_/CD_3_OD_,_ d, ppm): 5.80 (s, 1H, H-2), 6.80 (d, *J* = 3.0 Hz, 2H, H-2’/6’), 6.40 (t, *J* = 3.0 Hz, 1H, H-4’), 7.22-7.15 (m, 5H, H-2’’/3’’/4’’/5’’/6’’), 8.79 (bs, -OH), 5.80 (bs, 1H, -OH); ^13^C-NMR (100 MHz, CDCl_3_/CD_3_OD, d, ppm): 199.26 (C-1), 75.92 (C-2), 135.31 (C-1’), 107.91 (C-2’), 157.95 (C-3’), 108.54 (C-4’), 157.95 (C-5’), 107.91 (C-6’), 138.53 (C-1’’), 127.66 (C-2’’), 129.05 (C-3’’), 128.59 (C-4’’), 129.05 (C-5’’), 127.66 (C-6’’); Positive LC-QTOF-MS *m/z* (%): [M+K-H]^+^ 282.2693(67), calc. 282.2670.

**Compound 4 (1,2-Bis(3-hydroxyphenyl)-2-hydroxyethanone):** Yield: 68%; R_f _= 0.45 (chloroform-ethyl acetate-acetic acid: 25:10:1); FT-IR (cm^–1^): 3320, 2946, 1678, 1586, 1486, 1452, 1277, 1234, 1069, 1016, 996, 876, 779; ^1^H-NMR (400 MHz, CDCl_3_/CD_3_OD_,_ d, ppm): 5.84 (s, 1H, H-2), 6.79 (s, 1H, H-2’), 6.70-6.67 (m, 1H, H-4’), 7.36-7.31 (m, 2H, H-5’/5’’), 7.14 (d, 1H, *J* = 8.0 Hz, H-6’), 6.77 (s, 1H, H-2’’), 6.96-6.94 (m, 1H, H-4’’), 7.05 (d, *J* = 8.0 Hz, 1H, H-6’’), 5.07 (bs, 2-OH); ^13^C-NMR (100 MHz, CDCl_3_/CD_3_OD, d, ppm): 199.15 (C-1), 75.89 (C-2), 134.66 (C-1’), 115.87 (C-2’), 156.92 (C-3’), 115.40 (C-4’), 130.31 (C-5’), 121.47 (C-6’), 139.83 (C-1’’), 114.61 (C-2’’), 156.83 (C-3’’), 119.28 (C-4’’), 129.83 (C-5’’), 120.77 (C-6’’). 

**Compound 5 (2-Hydroxy-2-(3-hydroxyphenyl)-1-(4-hydroxyphenyl)ethanone):** Yield: 39%; R_f_ = 0.46 (chloroform-ethyl acetate-acetic acid: 25:10:1); light yellow oil; FT-IR (cm^–1^): 3292, 3045, 2211, 1740, 1668, 1590, 1514, 1453, 1239, 1171, 982; ^1^H-NMR (400 MHz, CD_3_OD_,_ d, ppm): 5.98 (s, 1H, H-2), 7.89 (d, *J* = 8.0 Hz, 2H, H-2’/6’), 6.80 (d, *J* = 8.0 Hz, 2H, H-3’/5’), 6.89 (s, 1H, H-2’’), 6.73 (d, *J* = 8.0 Hz, 1H, H-4’’), 7.14 (t, *J* = 8.0 Hz, 1H, H-5’’), 6.90 (d, *J* = 8.0 Hz, 1H, H-6’’), 5.21 (bs, -OH); ^13^C-NMR (100 MHz, CD_3_OD, ppm): 197.43 (C-1), 75.42 (C-2), 125.89 (C-1’), 131.46 (C-2’), 115.00 (C-3’), 162.60 (C-4’), 115.00 (C-5’), 131.46 (C-6’), 140.96 (C-1’’), 114.20 (C-2’’), 157.41 (C-3’’), 115.18 (C-4’’), 129.75 (C-5’’), 118.90 (C-6’’); Positive LC-QTOF-MS *m/z* (%): [M+K+Na+CH_3_OH-H]^+^ 337.2229(85), calc.337.2214.

**Compounds 6a and 6b (2-Hydroxy-1-(3,5-dihydroxyphenyl)-2-(3-hydroxyphenyl)ethanone)** **and (2-Hydroxy-1-(3-hydroxyphenyl)-2-(3,5-dihydroxyphenyl)ethanone):** Yield: 55%; R_f_ = 0.45 (chloroform-ethyl acetate-acetic acid: 25:10:1). Mix. m.p. (^o^C): 110-112; UV (MeOH) λ max nm (logɛ):210 (4,28); FT-IR (cm^–1^): 3363, 2915, 1682, 1600, 1457, 1339, 1283, 1165, 999, 722; ^1^H-NMR (400 MHz, CDCl_3_/(CD_3_)_2_CO, d, ppm): 7.89-6.70 (m, 14H, Ar-H), 6.10, 5.98 (s, s 1H/1H, 2x H-2); 9.60 (bs, Ar-OH); ^13^C-NMR (100 MHz, CDCl_3_/(CD_3_)_2_CO, d, ppm): 198.73, 193.62 (C=O), 77.78, 75.76 (C-2), 165.77, 158.51, 158.25, 158.19, 157.40, 156,94 136.26, 135.72, 135.40, 134.88, 130.80, 130.41 (Ar-C), 130.01, 129.67, 129.44, 121.10, 120.97, 120.74, 120.61, 120.14, 119.78, 116.39, 108.33, 107.56, 107.28, 103.79 (Ar-CH); Positive LC-QTOF-MS *m/z* (%): [M-H_2_O+CH_3_OH]^+^ 274.2644(100), calc. 274.2647.

**Compound 7 (1,2-Bis(3,5-dihydroxyphenyl)-2-hydroxyethanone):** Yield: 65%; R_f_ = 0.35 (chloroform-ethyl acetate-acetic acid: 25:10:1); light brown oil; UV (MeOH) λ max nm (logɛ):220(3,40); FT-IR (cm^-1^): 3360, 3160, 3037, 2917, 1687, 1594, 1453, 1343, 1306, 1166, 1006, 951, 707.; ^1^H-NMR (400 MHz, CDCl_3_/CD_3_OD_,_ d, ppm): 5.75 (s, 1H, H-2), 6.80 (d, *J* = 3.0 Hz, 2H, H-2’/6’), 6.40 (bs, 1H, H-4’), 6.31 (d, *J* = 3.0 Hz, 2H, H-2’’/6’’), 6.22 (bs, 1H, H-4’’); ^13^C-NMR (100 MHz, CDCl_3_/CD_3_OD, d, ppm): 199.02 (C-1), 75.87 (C-2), 136.08 (C-1’), 107.49 (C-2’), 158.62 (C-3’), 108.29 (C-4’), 158.62 (C-5’), 107.49 (C-6’), 141.33 (C-1’’), 106.44 (C-2’’), 158.50 (C-3’’), 102.90 (C-4’’), 158.50 (C-5’’), 106.44 (C-6’’); Positive LC-QTOF-MS *m/z* (%): [M+CO_2_-H_2_O+2H]^+^ 304.2571(85), calc. 304.2500, [M+CO]^+^ 304.2526(80), calc. 304.2500.

**Synthesis of hydroxy benzils (8-14):** Hydroxy benzoins (100–400 mg) were dissolved in acetone (5 mL) and conc. HNO_3_ (2-3 mL) was added, and the reactions were stirred at 50–70 ^°^C for 30–120 min [31]. The reactions were terminated after the TLC control. Acetone was evaporated, then water (30 mL) was added to the flask, extracted with ethyl acetate (3×30 mL) to give crude mixture then compounds **8-15** were purified with repeated VLC (Silica gel 230–400 mesh) using the increasing polarity of *n* -hexane, chloroform, ethyl acetate, and methanol solvent mixtures, and the fractions were checked by TLC (Figure). The synthesis of compounds **8** [23], **9** [9], **11** [44], and **15** [commercial product] had been mentioned in the literature.

**Compound 8 (1-(3-Hydroxyphenyl)-2-phenylethane-1,2-dione):** Yield: 25%;R_f_ = 0.55 (chloroform-ethyl acetate-acetic acid: 25:10:1); light yellow oil; FT-IR (cm^–1^): 3396, 2933, 1671, 1597, 1450, 1303, 1263, 1176, 942, 840, 780, 749, 635; ^1^H-NMR (400 MHz, CD_3_OD, d, ppm): 7.55 (d, *J* = 7.8 Hz, 1H, H-6’), 7.35 (m, 1H, H-5’), 7.28 (s, 1H, H-2’), 7.13 (m, 1H, H-4’); 7.91 (d, *J* = 8.0 Hz, 2H, H-2’’/6’’), 7.54 (t, *J* = 8.0 Hz, 2H, H-3’’/5’’), 7.69 (t, *J* = 8.0 Hz, 1H, H-4’’);^ 13^C-NMR (100 MHz, CD_3_OD, d, ppm): 196.58 (C-1), 202.50 (C-2), 134.40 (C-1’), 116.36 (C-2’), 159.60 (C-3’), 122.42 (C-4’), 131.53 (C-5’), 123.62 (C-6’), 135.62 (C-1’’), 130.81 (C-2’’), 130.40 (C-3’’), 136.30 (C-4’’), 130.40 (C-5’’), 130.81 (C-6’’).

**Compound 9 (1-(4-Hydroxyphenyl)-2-phenylethane-1,2-dione):** Yield: 16%; R_f_ = 0.53 (chloroform-ethyl acetate-acetic acid: 25:10:1); light yellow oil; FT-IR (cm^-1^): 3368, 3027, 2927, 2856, 1740, 1678, 1599, 1582, 1448, 1369, 1267, 1213, 1164, 1043, 879, 719, 611; ^1^H-NMR (400 MHz, CDCl_3_/(CD_3_)_2_CO, d, ppm): 7.83 (d, *J* = 8.0 Hz, 2H, H-2’/6’), 6.90 (d, *J* = 8.0 Hz, 2H, H-3’/5’), 7.94 (d, *J* = 8.0 Hz, 2H, H-2’’/6’’), 7.47 (t, *J* = 8.0 Hz, 2H, H-3’’/5’’), 7.62 (t, *J* = 8.0 Hz, 1H, H-4’’); ^13^C-NMR (100 MHz, CDCl_3_/(CD_3_)_2_CO, d, ppm): 193.49 (C-1), 195.35 (C-2), 125.15 (C-1’), 132.72 (C-2’), 116.15 (C-3’), 163.40 (C-4’), 116.15 (C-5’), 132.72 (C-6’), 133.12 (C-1’’), 129.88 (C-2’’), 128.98 (C-3’’), 134.83 (C-4’’), 128.98 (C-5’’), 129.88 (C-6’’).

**Compound 10 (1-(3,5-Dihydroxyphenyl)-2-phenylethane-1,2-dione):** Yield: 35%; R_f_ = 0.48 (chloroform-ethyl acetate-acetic acid: 25:10:1); light brown oil; FT-IR (cm^-1^): 3434, 2964, 1747, 1598, 1450, 1368, 1227, 1166, 1035; UV (MeOH) λ max nm (logɛ): 220(3,40); ^1^H-NMR (400 MHz, CDCl_3_/ (CD_3_)_2_CO, d, ppm): 6.94 (d, *J* = 3.0 Hz, 2H, H-2’/6’), 7.35 (bs, 1H, H-4’); 7.92 (d, *J* = 8.0 Hz, 2H, H-2’’/6’’), 7.50 (t, *J* = 8.0 Hz, 2H, H-3’’/5’’), 7.65 (t, *J* = 8.0 Hz, 1H, H-4’’), 9.08 (bs, -OH); ^13^C-NMR (100 MHz, CDCl_3_/ (CD_3_)_2_CO, d, ppm): 195.04 (C-1), 195.15 (C-2), 132.96 (C-1’), 108.30 (C-2’), 158.88 (C-3’), 110.04 (C-4’), 158.88 (C-5’), 108.30 (C-6’), 134.42 (C-1’’), 129.75 (C-2’’), 128.95 (C-3’’), 134.77 (C-4’’), 128.95 (C-5’’), 129.75 (C-6’’); Positive LC-QTOF-MS *m/z* (%): [M+Na+K]^+^ 304.2539(100), calc. 304.2580; [M+K+H]^+ ^282.2722(100), calc. 282.2753.

**Compound 11 (1,2-Bis(3-hydroxyphenyl)ethane-1,2-dione):** Yield : 45%; R_f_ = 0.45 (chloroform-ethyl acetate-acetic acid: 25:10:1); FT-IR (cm^–1^): 3380, 2960, 2931, 2874, 1736, 1646, 1618, 1582, 1452, 1350, 1225, 1194, 1108, 983, 865, 785, 684; ^1^H-NMR (400 MHz, (CD_3_)_2_CO, d, ppm): 7.72 (m, 6H-4’/4’’/5’/5’’/6’/6’’), 7.25 (m, 2H, H-2’/2’’), 9.00 (bs, 2H, -OH); ^13^C-NMR (100 MHz, (CD_3_)_2_CO, d ppm): 194.95 (C-1/2) 134.32 (C-1’/1’’), 115.03 (C-2’/2’’), 158.10 (3’/3’’), 121.25 (4’/4’’), 130.55 (5’/5’’), 122.41 (6’/6’’).

**Compound 12 (1-(4-Hydroxyphenyl)-2-(3-hydroxyphenyl)ethane-1,2-dione:** Yield: 23%;R_f_ = 0.40 (chloroform-ethyl acetate-acetic acid: 25:10:1); m.p. (^o^C): 60–62; UV (MeOH) λ max nm (logɛ): 203(4,03); FT-IR (cm^-1^): 3436, 2947, 1751, 1598, 1450, 1369, 1232, 1166, 1034; ^1^H-NMR (400 MHz, (CD_3_)_2_CO, d, ppm): 7.05 (s, 1H, H-2’), 7.38–7.24 (m, 2H, H-4’, 6’), 7.22 (t, 1H, J = 7.8 Hz, H-5’), 7.55/7.51 (s, s, 1H/1H, H-2’’, 6’’), 6.70/6.66 (s, s, 1H/1H, H-3’’, 5’’), 5.14 (bs, -OH); ^13^C-NMR (100 MHz, (CD_3_)_2_CO, d, ppm): 200.15 (C-1/2), 163.74, 157.64, 135.73, 129.73 (Ar-C), 149.93, 144.61, 132.15, 129.73, 126.36, 119.89, 117.64, 115.54, 114.19 (Ar-CH), 191.58 (-CHO); Positive LC-QTOF-MS *m/z* (%): [M+CH_3_OH]^ + ^274.2679(90), calc. 274.2695.

**Compound 13 (1-(3,5-Dihydroxyphenyl)-2-(3-hydroxyphenyl)ethane-1,2-dione):** Yield: 18%; R_f_ = 0.42 (chloroform-ethyl acetate-acetic acid: 25:10:1); light brown oil; UV (MeOH) λ max nm (logɛ): 211(4,34); FT-IR (cm^-1^): 3372, 2957, 1675, 1603, 1453, 1279, 1245, 1171; ^1^H-NMR (400 MHz, CDCl_3_/ (CD_3_)_2_CO), d, ppm): 7.15 (d, *J* = 3.0 Hz, 2H, H-2’, 6’), 6.93 (dd, *J* = 3.0/3.0Hz, 1H, H-4’), 7.68-7.25 (m, 4H, H-2’’,4’’,5’’,6’’), 8.86/8.75 (bs, 3x Ar-OH); ^13^C-NMR (100 MHz, CDCl_3_/ (CD_3_)_2_CO), d, ppm): 191.67 (C-1), 191.95 (C-2), 133.90 (C-1’), 107.97 (C-2’), 158.32 (C-3’), 109.31 (C-4’), 158.32 (C-5’), 107.97 (C-6’), 134.35 (C-1’’), 115.26 (C-2’’), 157.22 (C-3’’), 121.19 (C-4’’), 129.75 (C-5’’), 122.00 (C-6’’); Positive LC-QTOF-MS *m/z* (%): [M-H_2_O+CH_3_OH+2H]^+^ 274.2711(100), calc. 274.2720.

**Compound 14 (1,2-Bis(3,5-dihydroxyphenyl)ethane-1,2-dione):** Yield: 28%;R_f_ = 0.38 (chloroform-ethyl acetate-acetic acid: 25:10:1); FT-IR (cm^-1^): 3369, 2938, 1726, 1602, 1366, 1267, 1221, 1165, 1034; ^1^H-NMR (400 MHz, CDCl_3_/ (CD_3_)_2_CO, d, ppm): 6.85 (s, 4H, H-2’/6’/2’’/6’’), 6.69 (s, 2H, H-4’/4’’); ^13^C-NMR (100 MHz, CDCl_3_/ (CD_3_)_2_CO, d, ppm): 195.48 (C-1/2), 134.79 (C-1’/1’’), 107.45 (C-2’/6’/2’’/6’’), 159.58 (C-3’/5’/3’’/5’’), 109.39 (C-4’/4’’). 

**Synthesis of benzoin/benzil-D-glucosides (15-25):** Hydroxy benzoins (100-150 mg each, **1-7** ) or benzils (100-200 mg each, **8-14** ) were dissolved in anhydrous methanol (10 mL) under the inert nitrogen atmosphere. KOH (2-4 equiv.) dissolved in methanol (5 mL) and added to the reaction mixtures, which were stirred in an ice bath for half an hour. Then, tetra-O-acetyl- *α* -D-bromoglucose (TABG, 4 equiv.) in acetone was added to the reaction medium and stirred at room temperature for 12 h [36–38]. As a result of the TLC control of the reactions, NaOMe (5 equiv.) was added to the medium, and the reactions were terminated after 12–24 h with the control of TLC. Excess of NaOMe was killed by the addition of MeOH. The solvent was evaporated, then water (15 mL) was added to the flask, extracted with ethyl acetate (3×20 mL) to give crude mixture then compounds **15-25** were purified with repeated VLC (Silica gel 230–400 mesh) using the increasing polarity of *n* -hexane, chloroform, ethyl acetate, and methanol solvent mixtures, and the fractions were checked by TLC (Figure).

**Compound 15 (2-Hydroxy-1-(3-O-** β **-D-glucopyranosylphenyl)-2-phenylethanone):** Yield: 15%; diastereomer; R_f_ = 0.5 (chloroform-methanol: 8:2); light yellow oil; UV (MeOH) λ max nm (logɛ): 210(4,56); FT-IR (cm^–1^): 3342, 3020, 2924, 1676, 1641, 1596, 1448, 1400, 1256, 1072, 1040, 892; ^1^H-NMR (400 MHz, (CD_3_)_2_CO, d, ppm): 7.67–7.21 (m, 18H, Ar-H), 6.13, 6.12 (m, 2H, H-2/H-2), 5.09 (d, *J* = 7.6 Hz, 1H, Glu H-1), 5.01(d, *J* = 7.6 Hz, 1H, Glu H-1), 4.75-3.22 (m, 12H, glucose H2-H6); ^13^C-NMR (100 MHz, (CD_3_)_2_CO, d, ppm): 191.83 (C = O), 163.53, 144.58, 133,81, 133,49 (Ar-C), 131.43, 130.21, 129,64, 128.65, 123.29, 122.87, 122.53, 121.02, 117.55, 116.58 (Ar-CH), 101.23, 101.03 (anomeric CH), 76.95, 76.92 (C-2), 77.0, 73.80, 73.76, 70.73 (glucose CH), 61.67 (glucose CH_2_). Positive LC-QTOF-MS *m/z* (%): [M+Na]^+^ 413.2563(20), calc. 413.2549.

**Compound 16 (2-Hydroxy-1-(4-O-** β **-D-glucopyranosylphenyl)-2-phenylethanone):** Yield: 17%; diastereomer; R_f_ = 0.68 (chloroform-methanol: 8:2); light yellow oil; UV (MeOH) λ max nm (logɛ): 210(5,43); FT-IR (cm^–1^): 3374 3018, 2927, 1582, 1410, 1348, 1313,1160, 1078, 1048, 610; ^1^H-NMR (400 MHz, CD_3_OD, d, ppm): 7.64 (bd, 4H, H-2’/H-6’), 6.40 (bd, 4H, H-3’/H-5’), .7.42–7.10 (m, 10H, H-2’’-6’’), 4.80 (anomeric CH remained within the water peak), 4.62-3.12 (m, 12H, glucose H2-H6); ^13^C-NMR (100 MHz, CD_3_OD, d, ppm): 196.29 (C=O), 165.07, 140.89, 119.28 (Ar-C), 131.98, 128.35, 127.63, 127.39, 118.76 (Ar-CH), 103.99 (anomeric CH), 76.53 (benzoin CH), 76.41, 74.41, 73.60, 69.99 (glucose CH), 60.69 (glucose CH_2_). Positive LC-QTOF-MS *m/z* (%): [M+Na]^+^ 413.1121(9), calc. 413.1141; [M+Na-H]^+^ 412.1015(23), calc. 412.1063.

**Compound 17 (2-Hydroxy-1-(3,5-Di-O-** β **-D-glucopyranosylphenyl)-2-phenylethanone):** Yield: 14%; diastereomer (2:1); R_f_ = 0.74 (chloroform-methanol: 8:2); light yellow oil; UV (MeOH) λ max nm (logɛ): 211(3,52); FT-IR (cm^–1^): 3367, 2972, 2270, 1720, 1269, 1057; ^1^H-NMR (400 MHz, (CD_3_)_2_CO, d, ppm): 8.05-6.48 (m, 16H, Ar-H), 6.06/6.00 (s, s, 1H, 1H, H-2/H-2), 5.03 (d, *J* = 7.6 Hz, 1H, Glu H-1), 4.98 (d, *J* = 7.6 Hz, 1H, Glu H-1), 4.46–3.32 (m, 24H, glucose H2-H6); ^13^C-NMR (100 MHz, (CD_3_)_2_CO, d, ppm): 199.00 (C=O), 159.02, 158.57, 141.90, 135.09 (Ar-C), 129.46, 129.02, 128.72, 128.65, 127.64, 127.52, 110.16, 109.95, 108.21, 108.02 (Ar-CH), 100.89/100.83 (anomeric CH), 76.90, 76.84, 76.06, 74.12, 73.84, 73.65, 70.38, 70.17 (benzoin CH and glucose CH), 63.56/63.38 (glucose CH_2_); Positive LC-QTOF-MS *m/z* (%): [M+K-CH_3_OH-2H]^+^ 573.1286(100), calc. 573.1249.

**Compounds 18a+b (2-Hydroxy-1-(3-O-** β **-D-glucopyranosylphenyl)-2-(3-hydroxyphenyl)-ethanone; 2-Hydroxy-1-(3-hydroxyphenyl)-2-(3-O-** β **-D-glucopyranosylphenyl)ethanone):** Yield: 11%; diastereomer (2:1); R_f_ = 0.80 (chloroform-methanol: 8:2); light yellow oil; UV (MeOH) λ max nm (logɛ): 220(3,45); FT-IR (cm^–1^): 3343, 3030, 2923, 1636, 1586, 1447, 1397, 1251, 1067, 1033, 1014, 892, 786; ^1^H-NMR (400 MHz, CD_3_OD, d, ppm): 7.48–6.55 (m, 32H, Ar-H), 6.00, 5.95 (m, 4H, H-2/H-2), 4.84–4.74 (anomeric CH remained within the water peak), 4.43-3.17 (m, 24H, glucose H2-H6); ^13^C-NMR (100 MHz, CD_3_OD, d, ppm): 198.97, 198.94, 198.73, 198.50 (C=O), 157.80, 157.59, 157.54, 157.44, 140.64, 140.59, 140.37, 140.32, 135.97, 135.86, 135.84, 135.74 (Ar-C), 129.75-114.29 (Ar-CH), 103.93, 103.78, 103.1, 100.64 (anomeric -CH), 76.45, 76.37, 76.25, 76.13, 73.98, 73.63, 73.65, 73.56, 70.65, 70.13 (benzoin CH and Glucose CH (C-2-5)), 63.45, 63.38 (Glucose -CH_2_OH); Positive LC-QTOF-MS m/z (%): [M+C_6_H_12_O_6_-CH_3_OH-H]^+^ 563.5404(100), calc. 563.5404.

**Compound 19 (2-Hydroxy-1-(4-O-** β **-D-glucopyranosylphenyl)-2-(3-O-** β **-D-glucopyranosyl-phenyl)ethanone):** Yield: 18%; diastereomer (1:2); R_f_ = 0.45 (chloroform-methanol: 8:2); light yellow oil; UV (MeOH) λ max nm (logɛ): 213(4,58); FT-IR (cm^-1^): 3380, 3032, 2924, 1734, 1596, 1450, 1376, 1250, 1053; ^1^H-NMR (400 MHz, CD_3_OD, d, ppm): 8.02 (d, *J* = 7.8 Hz, 8H, H-2’/6’), 7.34 (d, *J* = 7.8 Hz, 8H, H-3’/5’), 7.80-7.45 (m, 16H, H-2’’,4’’,5’’,6’’), 6.11, 6.08 (s, s, 1H, 1H, H-2/H-2), 5.13–5.08 (anomeric CH beside the water peak), 4.52–3.38 (m, 48H glucose CH and CH_2_); ^13^C-NMR (100 MHz, CD_3_OD, d, ppm): 193.74, 192.80 (C=O), 76.50, 76.03 (C-2), 164.01, 159.30, 139.28, 114.59 (Ar-C), 130.28, 129.8, 122.25, 122.15, 122.06, 119.88, 116.14 (Ar-CH), 102.05, 101.36 (anomeric -CH), 77.90, 74.63, 74,55, 73.45, 73.33, 73.21, 69.89, 69.67, 69.54, 68.52 (Glucose C2-C5), 62.92, 60.26 (Glucose -CH_2_); Positive LC-QTOF-MS *m/z* (%): [M+Na]^+ ^575.2733(75), calc. 575.2740.

**Compound 20 (2-Hydroxy-1-(3,5-di-O-** β **-D-glucopyranosylphenyl)-2-(3-O-** β **-D-glucopyranosylphenyl)ethanone):** Yield: 12%; diastereomer; R_f_ = 0.5 (chloroform-methanol: 8:2); light yellow oily; UV (MeOH) λ max nm (logɛ): 213(4,03); FT-IR (cm^–1^): 3385, 3028, 2923, 2568, 1688, 1597, 1456, 1287, 1075, 1034; ^1^H-NMR (400 MHz, CD_3_OD, d, ppm): 7.74-6.87 (m, 16H, Ar-H), 5.84, 5.71 (s, s, benzoin -CH), 5.04–4.8 (anomeric CH beside the water peak), 4.65-3.30 (m, 36H, glucose CH and CH_2_); ^13^C-NMR (100 MHz, CD_3_OD, d, ppm): 198.73, 194.24 (C=O), 76.74 (C-2), 166.90, 160.50, 158.48, 158.27, 158.20, 157.89, 157.84 139.25, 138.64, 134.41, 134.28, 131.85, 131.22 (Ar-C), 131.22, 129.88, 129.63, 124.10, 123.65, 120.32, 120.11, 120.08, 119.87, 116.59, 116.20, 116.06, 109.33, 109.16, 108.78 (Ar-CH), 103.97, 100.72 (anomeric CH), 76.48, 76.16, 74.98, 72.56, 69.81 (Glucose CH (C-2-5)), 61.31, 61.01 (glucose CH_2_); Positive LC-QTOF-MS *m/z* (%): [M+Na-H_2_O-H]^+^ 750.5818(74), calc. 750.5836; [M+Na-CH_3_OH-H]^+^ 736.5696(100), calc. 736.5600.

**Compound 21 (1-(3-O-** β **-D-Glucopyranosylphenyl)-2-phenylethane-1,2-dione):** Yield: 18%; R_f_ = 0.77 (chloroform-methanol: 8:2); light yellow oil; UV (MeOH) λ max nm (logɛ): 205(4,61);FT-IR (cm^–1^): 3364, 2938, 1739, 1665, 1445, 1227, 1074; ^1^H-NMR (400 MHz, (CD_3_OD, d, ppm): 7.96-7.31 (m, 9H, Ar-H), 4.99 (d, *J =* 7.6 Hz, anomeric CH), 4.49-3.33 (m, 6H, glucose CH and CH_2_); ^13^C-NMR (100 MHz, (CD_3_OD, d, ppm): 194.51, 192.42 (C=O), 158.10, 134.94, 132.82 (Ar-C), 134.16, 130.07, 129.39, 128.98, 124.20, 123.60, 115.90 (Ar-CH), 100.68 (anomeric CH), 76.26, 74.12, 73.32, 70.21 (glucose CH), 63.42 (glucose CH_2_); Positive LC-QTOF-MS *m/z* (%): [M-2CH_3_OH+H]^+^ 325.2283(100), calc. 325.2280.

**Compound 22 (1-(4-O-** β **-D-Glucopyranosylphenyl)-2-phenylethane-1,2-dione):** Yield: 12%;R_f_ = 0.77 (chloroform-methanol: 8:2); light yellow oil; UV (MeOH) λ max nm (logɛ): 210(3,92); FT-IR (cm^-1^): 3385, 2972, 1710, 1603, 1445, 1270, 1058; ^1^H-NMR (400 MHz, ((CD_3_)_2_CO, d, ppm): 7.94 (m, 4H, H-2’,6’, H-2’’,6’’), 7.63 (t, *J* = 7.6 Hz, 2H, H-3’’, 5’’), 7.74 (t, *J* = 7.7 Hz, 1H, H4’’), 7.22 (d, *J* = 7.8 Hz, 2H, H-3’, 5’), 5.20 (d, *J =* 7.6 Hz, 1H, Glu H-1), 4.43-3.43 (m, 6H, glucose H2-H6); ^13^C-NMR (100 MHz, ((CD_3_)_2_CO, d ppm): 198.00, 194.58 (C=O), 161.59, 133.36, 126.93 (Ar-C), 134.98, 131,88, 129.57, 129.24, 116.72 (Ar-CH), 100.11 (anomeric CH), 76.80, 74.26, 73.58, 70.15 (glucose CH), 63.24 (glucose CH_2_); Positive LC-QTOF-MS *m/z* (%): [M+K+Na+3H]^+^ 453.1011(100), calc. 453.1016.

**Compound 23 (1-(3,5-Di-O-** β **-D-glucopyranosylphenyl)-2-phenylethane-1,2-dione):** Yield: 9%; R_f_ = 0.75 (chloroform-methanol: 8:2); light yellow oil; UV (MeOH) λ max nm (logɛ): 215(4,03); FT-IR (cm^–1^): 3627, 2975, 2256, 1713, 1524, 1386, 1058; ^1^H-NMR (400 MHz, (CD_3_)_2_CO, d, ppm): 7.80 (d, *J* = 7.8 Hz, 2H, H-2’’, 6’’), 7.80-7.40 (m, 3H, H-3’’, 4’’, 5’’), 7.08 (bs, 2H, H-2’, 6’), 6.95 (bs, 1H, H4’), 4.98 (d, *J =* 7.6 Hz *,* Glu H-1), 4.30-3.44 (m, 6H, glucose H2-H6); ^13^C-NMR (100 MHz, (CD_3_)_2_CO, d, ppm): 194.98, 193.68 (C=O), 159.27, 134.73, 133.96 (Ar-C), 135.21, 129.55, 129.45, 129.33, 124.71, 110.25, 109.18 (Ar-CH), 101.05 (anomeric CH), 77.08, 73.67, 70.21 (glucose CH), 61.57 (glucose CH_2_); Positive LC-QTOF-MS *m/z* (%): [M-CH_3_OH-CO_2_-3H]^+^ 325.2162(100), calc. 325.2162.

**Compound 24 (1-(3-O-** β **-D-Glucopyranosylphenyl)-2-(3-hydroxyphenyl)ethane-1,2-dione):** Yield: 42%; R_f_ = 0.75 (chloroform-methanol: 8:2); light yellow oil; UV (MeOH) λ max nm (logɛ): 210(4,67); FT-IR (cm^–1^): 3364, 2938, 1739, 1665, 1445, 1227, 1075; ^1^H-NMR (400 MHz, (CD_3_OD, d, ppm): 7.78-6.77 (m, 8H, H-2’,4’,5’,6’, H-2’’,4’’,5’’,6’’), 4.97 (d, *J =* 7.8 Hz *,* anomeric CH), 3.96-3.17 (m, 6H, glucose CH and CH_2_); ^13^C-NMR (100 MHz, (CD_3_OD, d, ppm): 193.42, 193.35, (C=O), 156.56, 135.01, 134.50, 132.58 (Ar-C), 128.54, 128.60, 126.72, 123.45, 125.82, 121.65, 120.91, 114.28 (Ar-CH), 102.43 (anomeric -CH), 75.20, 74.96, 72.10, 68.68 (glucose CH), 59.77 (glucose CH_2_); Positive LC-QTOF-MS *m/z* (%) : [M+Na-H]^+^ 588.4345(100), calc. 588.4387.

**Compound 25 (1-(3-O-** β **-D-Glucopyranosylphenyl)-2-(4-hydroxyphenyl)ethane-1,2-dione):**

Yield: 12%; R_f_ = 0.60 (chloroform-methanol: 8:2); light yellow oil; UV (MeOH) λ max nm (logɛ): 206(4,97); FT-IR (cm^-1^): 3748, 3620, 2973, 2302, 1732, 1386, 1228, 1057; ^1^H-NMR (400 MHz, (CD_3_OD, d, ppm): 7.42 (s, 1H, H-2’), 7.38–7.24 (m, 2H, H-4’, 5’), 7.18 (d, 1H, *J* = 7.8 Hz, H-6’), 7.66/7.64 (s, s, 1H/1H, H-2’’, 6’’), 6.83/6.79 (s, s, 1H/1H, H-3’’, 5’’), 4.96 (d, *J =* 7.6 Hz *,* 1H, Glu H-1), 4.31–3.37 (m, 6H, glucose H_2_-H_6_); ^13^C-NMR (100 MHz, (CD_3_OD, d, ppm): 192.34, 191.98 (C=O), 158.20, 143.92, 135.94, 126.86 (Ar-C), 129.67, 129.18, 126.33, 122.95, 119.40, 117.31, 115.17, 114.28 (Ar-CH), 100.95 (anomeric CH), 76.90, 76.57, 73.51, 70.04 (glucose CH), 61.14 (glucose CH_2_); Positive LC-QTOF-MS *m/z* (%): [M+H+C_6_H_12_O_6_]^+^ 585.5184(15), calc. 585.5156.

### 2.1. Biological activities

#### 2.1.1. Antioxidant activity

Antioxidant activities of the synthetic compounds **1-25** were tested against iron (III) / ferric reducing antioxidant power (FRAP), Cu (II) reducing antioxidant capacity (CUPRAC), and 2,2-Diphenyl-1-picrylhydrazyl radical quenching capacity (DPPH) methods according to the literature [45–50] (Table 2). Butylated hydroxytoluene for DPPH and Trolox for CUPRAC and FRAP was used as standard.

**Table 2:** Antioxidant (FRAP, CUPRAC, and DPPH) activities of compounds 1-25.

Hydroxy Benzoin			
No	FRAPa	CUPRACb	DPPHc
1	1238 ± 34.7	738.33 ± 12.5	15.21 ± 2.1
2	1881 ± 75.5	140.00 ± 11.5	13.78 ± 1.3
3	1111 ± 47.9	90.00 ± 6.8	8.72 ± 0.26
4	1534 ± 75.0	398.33 ± 22.1	8.16 ± 0.3
5	2090 ± 101.4	1113.33 ± 64.9	8.12 ± 1.2
6a+b	2237 ± 58.3	506.67 ± 17.3	9.48 ± 0.3
7	1715 ± 96.8	95.00 ± 2.4	10.85 ± 0.7
Hydroxy Benzil			
8	1678 ± 64.6	1095.00 ± 18.1	13.56 ± 1.2
9	1946 ± 83.7	48.33 ± 5.5	8.64 ± 0.5
10	1830 ± 44.8	455.00 ± 10.1	52.10 ± 0.4
11	1340 ± 37.9	175.00 ± 3.5	10.03 ± 0.8
12	1555 ± 34.1	155.00 ± 1.0	12.87 ± 0.9
13	1844 ± 56.2	143.33 ± 7.6	9.42 ± 0.8
14	1974 ± 76.9	133.33 ± 10.9	7.38 ± 1.0
Benzoin-O-β-D-Glucoside			
15	2311.04 ± 31.21	141.74 ±28.35	107.60 ± 9.09
16	1956.25 ± 48.13	73.54 ± 17.36	44.75 ± 3.04
17	2647.92 ± 31.92	287.40 ± 9.81	8.22 ± 1.08
18a+b	2743.75 ± 25.20	385.80 ± 37.12	12.11 ± 0.43
19	2612.50 ± 37.04	421.74 ± 24.39	8.86 ± 0.37
20	2918.75 ± 36.14	775.58 ± 12.34	7.78 ± 0.12
Benzil-O-β-D-Glucoside			
21	1981.25 ± 28.33	109.94 ± 19.42	36.94 ± 1.71
22	1845.42 ± 41.36	248.97 ± 34.06	47.54 ± 1.05
23	2267.50 ± 24.14	273.67 ± 27.23	17.74 ± 0.35
24a,b	2401.25 ± 56.34	251.46 ± 43.08	25.40 ± 0.19
25	2570.42 ± 25.01	147.54 ± 25.04	26.33 ± 0.39
BHT	-	-	6.47 ± 0.12

aFRAP, the iron reducing antioxidant power (μg/mL trolox/gram DW), bCUPRAC, copper reducing antioxidant power (μg/mL trolox/gram DW), cDPPH, 2,2-diphenyl-1-picrylhydrazyl radical scavenging capacity (mg/mL), BHT: di-t-butylhydroxytoluene.

**Ferric reducing antioxidant power (FRAP) assay:** The method was carried out based on the determination of the iron ions reducing the samples’ power. First, 2,4,6-tripyridyl-s-triazine (31.2 mg, TPTz) was dissolved in a mixture of hydrochloric acid (50 μL) and distilled water (10 mL). Then, FeCl_3_ (32 mg) was dissolved in distilled water (10 mL). Finally, distilled water (250 mL) was added to acetic acid (4.1 mL, 80%), and sodium acetate (0.66 g) was completely dissolved in this solution. Buffer, TPTz, and FeCl_3_ were mixed at 10/1/1 ratios, and 2 mL of this mixture was mixed with 0.1 mL of compounds **1-25** (2 mg/mL) and incubated at 30^o^C for 30 min. As a standard, different concentrations of Trolox solution (15.63, 31.25, 62.5, 125, and 250 μg/mL) were used instead of the sample. At the end of the incubation, the samples’ absorbance was read at 595 nm, and the results are given as Trolox equivalents. Results were expressed as μmol Trolox/g dry weight of compounds **1-25** (μg/mL Trolox/g DW) [48,50] (Table 2). 

**Copper ions reducing activity (CUPRAC):** In a test tube, ammonium acetate (1 mL, 1 M), CuCl_2_ (1 mL, 10 mM), and neocuproin (1 mL, 7.5 mM) solutions were taken, and 0.5 mL of compounds **1** - **25** and standards (Trolox) at different concentrations (15.63, 31.25, 62.5, 125 and 250 μg/mL) were mixed, and 1 mL of distilled water was added to each tube. After 30 min in a dark environment at room temperature, it was read against blank at 450 nm using Shimadzu UV-1600 spectrophotometer [49], and results are given in Table 2.

**DPPH radical scavenging activity:** In vitro antioxidant properties of compounds, **1-25** were tested using 2,2-diphenyl-1-picrylhydrazyl scavenging (DPPH). 0.75 mL of compounds **1** - **25** and standard (BHT) at varying concentrations (mg/mL) and 0.75 mL of 0.1 mM DPPH solution were mixed. All tubes were left in the dark for 50 min at room temperature. The absorbance was read 517 nm using Shimadzu UV-1600 spectrophotometer, and results are given as SC_50_ value (mg/mL) in Table 2 [45–47].

#### 2.1.2. Microorganisms used for antimicrobial activity

The test microorganisms used in the study were obtained from Refik Saydam Hıfzısıhha Institute (Ankara, Turkey) and are as follows. *Escherichia coli* ATCC 25922 (Ec), *Yersinia pseudotuberculosis* ATCC911 (Yp), *Pseudomonas aeruginosa* ATCC27853 (Pa), *Staphylococcus aureus* ATCC25923 (Sa), *Streptococcus mutans* RSKK07038 (Sm), *Enterococcus faecalis* ATCC29212 (Ef), *Paenibacillus larvae* DSM7030 (PSP), *Bacillus cereus* Roma709 (Bc), *Bacillus subtilis* ATCC1266 (Bs), *Mycobacterium smegmatis* ATCC607 (Ms), *Candida albicans* ATCC60193 (Ca). Inhibition diameters were measured by the agar well diffusion method [51–53], and the MIC value was determined as microgram-milliliter (µg / mL) to the microdilution technics (Table 3). 

**Table 3:** Antimicrobial minimum inhibition concentrations of compounds 1-25.

No	Stock sol.µg/mL	Microorganism and minimum inhibition concentration (MIC, µg/mL)								
		Gram (-)			Gram (+)				Tub.	Mush.
		Ec	Yp	Pa	Sa	Sm	Ef	Bc	Ms	Ca
Hydroxy Benzoin										
1	26400	330	1320	-	-	-	-	1320	165	330
2	41300	1032	1032	2065	1032	1032	-	1032	129	516
3	66300	820	1657	207	207	414	820	207	103	414
4	51200	320	640	320	640	-	-	160	160	1280
5	62000	387	775	96	96	48	1550	48	48	-
6a+b	8300	103	103	52	12	12	24	24	12	103
7	22700	141	1135	283	283	-	141	141	70	283
Hydroxy Benzil										
8	13400	167	167	11	11	21	21	11	11	41
9	17400	108	217	108	108	-	108	108	108	108
10	1300	17	65	32	65	-	-	17	17	17
11	5800	36	145	18	9	18	-	18	9	36
12	1500	75	38	75	38	-	18	75	18	18
13	13400	167	167	11	11	21	21	11	11	41
14	4100	102	102	51	-	-	51	102	51	51
Amp.	10	10	18	>128	35	NT	10	NT	-	-
Strep.	10	-	-	-	-	-	-	-	4	-
Flu.	5	-	-	-	-	-	-	-	-	<8

Ec: E. coli, Yp: Y. pseudotuberculosis, Pa: P. aeruginosa, Sa: S. aureus, Sm: S. mutans, Ef: E. faecalis, Psp: P. larvae, Bc: B. cereus, Bs: B. suptilis, Ms: M. smegmatis, Ca: C. albicans, (-): no result. Amp.: Ampicillin, Str.: Streptomycin, Flu.: Fluconazole, NT; not tested. Tub.: Tuberculosis

**Antimicrobial activity assessment (agar-well diffusion method):** The antimicrobial screening test using the agar-well diffusion method as adapted was used earlier [53–54]. Each microorganism was suspended in Mueller-Hinton broth (Difco, Detroit, MI) and diluted approximately 10^6^ colony-forming units (CFU) per mL. They were “flood-inoculated” onto the surface of Mueller–Hinton agar, brain heart infusion agar, and potato dextrose agar (PDA) (Difco, Detriot, MI) and then dried. Brain heart infusion agar was used for *M. smegmatis* and *S. mutans* . For *C. albicans,* PDA was used. Five-millimeter diameter wells were cut from the agar using a sterile cork-borer, and 50 μL of the compound substances were delivered into the wells. The plates were incubated for 24–48 h at 36 °C. Antimicrobial activity was evaluated by measuring the zone of inhibition against the test organism. Compound stock solutions were prepared at different concentrations (1.100–80.200 μg/mL) according to the amount of material obtained. The 1/10 dilution of each solvent was used as a control.

**Minimal inhibition concentration (MIC) assay:** The antimicrobial properties of compounds **1-25** were investigated quantitatively in respective broth media by using the microdilution method, and the minimal inhibition concentration (MIC) values (μg/mL) were examined [53]. The antibacterial activity assays were carried out in Mueller–Hinton broth (MHB) at pH = 7.0±0.2 and 18–24 h at 36 °C incubated. For the antifungal activity test were used yeast extract peptone dextrose (YEPD) broth (pH = 6.5 ± 0.2) and 48 h at 36 °C incubated. Brain heart infusion broth (BHI) (Difco, Detriot, MI) was used for *M. smegmatis* and *S. mutans* and incubated for 72 h at 36 °C. The minimal inhibition concentration value was defined as the lowest concentration that showed no growth. Ampicillin (10 mg/mL), streptomycin (10 mg/mL) and fluconazole (5 mg/mL) were used as standard antibacterial and antifungal drugs, respectively (Table 3). The 1/10 dilution of each solvent was used as a control.

#### 2.1.3. Enzyme inhibitions

**Acetylcholinesterase (AChE) inhibition:** The acetylcholinesterase method is based on the principle that thiocholine released by a chromogenic reagent 5,5-dithio-bis-(2-nitrobenzoic acid) gives a colored product. The sample solution (10 μL) and acetylcholinesterase solution (20 μL) were mixed in Tris-HCl buffer (130 μL, pH 8.0). It was then incubated at 25 °C for 10 min in a 96-well microplate. Then, DTNB (20 μL) and acetylthiocholine iodide (20 μL) were mixed. Similarly, an enzyme-free tube was prepared blindly. Sample and blank absorbances were read after 10 min of incubation at 25 °C at 405 nm. Acetylcholinesterase inhibitory activity was given equivalent to galantamine [55], and the results were given in Table 4.

**Table 4:** Enzyme inhibition of compounds 1-25, IC50 (μg/mL).

Hydroxy Benzoin					
No	AChE	BChE	Tyrosinase	α-Amylase	α-Glucosidase
1	>1000	431.18	46.99	>1000	58.16
2	244.54	73.62	>1000	>1000	547.97
3	397.36	144.49	83.14	206.25	812.10
4	110.96	112.86	>1000	239.82	>1000
5	787.84	>1000	163.82	104.99	>1000
6a+b	>1000	592.92	25.45	230.76	76.53
7	122.40	>1000	130.44	97.51	>1000
Hydroxy Benzil					
8	>1000	>1000	>1000	>1000	>1000
9	144.01	>1000	>1000	219.82	47.78
10	38.90	>1000	54.44	>1000	100.08
11	62.16	339.49	>1000	349.71	66.700
12	501.53	462.11	>1000	>1000	54.69
13	>1000	>1000	>1000	>1000	>1000
14	70.39	>1000	634.91	>1000	87.10
Galantamine	9.27	33.73	-	-	-
Kojic acid	-	-	12.78	-	-
Acarbose	-	-	-	93.12	36.65
Benzoin-O-β-D-Glucoside					
15	11.97	>1000	>1000	251.48	>1000
16	155.85	188.83	>1000	>1000	>1000
17	>1000	>1000	>1000	>1000	193.40
18a+b	>1000	462.26	>1000	>1000	>1000
19	10.52	>1000	31.22	139.78	124.94
20	>1000	189.56	71.81	>1000	48.25
Benzil-O-β-D-Glucoside					
21	>1000	>1000	89.93	282.98	>1000
22	132.83	527.25	727.98	>1000	212.24
23	70.47	>1000	208.42	101.97	>1000
24	281.66	212.79	148.48	176.99	58.90
25	227.19	133.86	416.31	>1000	>1000
Galantamine	7.71	33.71	-	-	-
Kojic acid	-	-	15.68	-	-
Acarbose	-	-	-	85.83	36.87

**Butyryl cholinesterase (BChE) inhibition:** Butyrylcholinesterase inhibition is based on acetylcholine’s hydrolysis by cholinesterase to 5,5-dithio-bis-(2-nitrobenzoic acid) (DTNB) into yellow colored 5-thio-2-nitrobenzoic acid. The sample solution (10 μL) and butyrylcholinesterase solution (20 μL) were mixed in Tris-HCl buffer (130 μL, pH = 8.0). It was then incubated at 25 °C for 10 min in a 96-well. Similarly, an enzyme-free tube was prepared blindly. Sample and blank absorbances were read after 10 min of incubation at 25 °C at 405 nm. Butyrylcholinesterase inhibitory activity was given equivalent to galantamine [55], and results were given in Table 4.

**Tyrosinase inhibition:** Tyrosinase inhibitor activity was performed by the dopachrome method using L-DOPA as a substrate. The sample solution (25 μL) was mixed with tyrosinase solution (40 μL) and phosphate buffer (100 μL, pH 6.8) in a 96-well microplate and incubated at 25 °C for 15 min. The reaction was initiated by the addition of L-DOPA (40 μL). Similarly, the enzyme-free blank solution was prepared, and the sample and blank absorbance were read at 492 nm after incubating at 25 °C for 10 min. Tyrosinase inhibitory activity results were given as equivalent to kojic acid [56], and results were given in Table 4.

**α-Amylase inhibition:** α-Amylase inhibitor activity was applied using the Caraway-Somogyi iodine/potassium iodide (I_2_/KI) method. Sample solutions (25 μL) were mixed with the α-amylase solution (50 μL) in phosphate buffer (pH = 6.9, 6 mM sodium chloride) in a 96-well microplate. The mixture was incubated at 37 °C for 10 min. After pre-incubation, the reaction was initiated when the starch solution (50 μL, 0.05%) was added. Similarly, the enzyme-free blank solution was prepared. The reaction mixture was incubated for 10 min at 37 °C, and the reaction was stopped by adding HCl (25 μL, 1 M). Following this, iodine -potassium iodide solution (100 μL) was added. Sample and blank absorbance were read at 630 nm, and α-amylase inhibitor activity results were given as acarbose equivalent [57–58], and results were given in Table 4.

**α-Glucosidase inhibition:** α-Glucosidase inhibitor activity was applied according to the method of Palanisamy et al. Sample solution (50 μL), glutathione (50 μL), α-glucosidase solution (50 μL) phosphate buffer (pH = 6.8) and PNPG (4-Nitrophenyl *β* -D-glucuronide) (50 μL) solution mixed in a 96-well microplate. It was incubated at 37 °C for 15 min. Similarly, an enzyme-free blank was prepared. The reaction was stopped when sodium carbonate (50 μL, 0.2 M) was added. Sample and blank absorbance were read at 400 nm. α-Glucosidase inhibitor activity was given as acarbose equivalent [57–58], and results were given in Table 4.

#### 2.1.4. Anticancer activity

**Anticancer cell lines and cell culture:** The human cervical cancer cell line (HeLa) and human retinal normal cell line (RPE) were used to determine the anticancer activities of molecules synthesized within the scope of the project [59–61]. All cell preparation processes were carried out in a sterile environment in a laminar cabinet. HeLa cell line was used in DMEM medium supplemented with 10% FBS (Fetal Bovine Serum) and 2% PenStrep (Penicillin-Streptomycin) solution at 37 °C, 5% CO_2_ conditions, after achieving sufficient concentration (confluent). DMEM/F12 medium was used for RPE cell lines. Inoculation was carried out on the measurement plates with 10,000 cells per well. After approximately 16 h of pre-incubation, test molecules were added, and measurements were made after 24 h of incubation, and results were given in Table 5.

**Cell proliferation measurement, determination of GI** **_50_** **and IC** **_50_** **values:** MTT [3-(4,5-dimethyl-thiazol-2-yl)-2,5-diphenyl tetrazolium bromide] test was used to measure the effects of synthesized test compounds **4** , **12** , **18a+b** , and **25** on cell proliferation (IC_50_ and GI_50_ values). This test protocol was applied after the cancer cell lines were incubated for 24 h with test substances. The results were reported as % cell inhibition, with the solvent’s optical density (DMSO) treated cells considered to be 100%. Accordingly, the % inhibition was calculated according to the formula [1-(test substance A/solvent control A)×100. MTT on cells of increasing concentrations (1, 2, 4, 8, 16, 32, 64, and 128 µg / mL) of each test substance over a certain range to determine the IC_50_ concentrations of test substances (concentration that inhibits the proliferation of 50% of cells in the medium) were prepared. Results were analyzed using a logarithmic function with the help of the Excel program over the logarithmic curve prepared from the absorbance values obtained after the test (Table 5). The following formula is used for the GI_50_ value calculation as cell proliferation: [(Ti-Tz)/(C-Tz)]x100 if Ti>/=Tz (cytostatic effect) or [(Ti-Tz)/Tz]x100 if Ti<Tz (cytotoxic or cytotoxic effect) (Tz; zero points, C; control growth, Ti; inhibition caused by test substance). GI_50_: Concentration value that reduces growth by 50% ([(Ti-Tz)/(C-Tz)]×100 = 50). The following formula is used for the IC_50_ value calculation. Accordingly, the % inhibition was calculated according to the formula [1-(A test substance/A solvent control)×100 [59-61].

**Table 5:** Antiproliferative effects of compounds 4, 12, 18a+b, and 25 on HeLa and RPE normal cell lines, µg/mL.

Compounds	4	12	18a+b	25	Cisplatin
HeLa cell line					
GI50	1.12	2.04	2.23	2.21	1.13
IC50	42.67	85.17	104.49	81.49	42.76
RPE normal cell line					
GI50	1.15	1.92	1.90	3.38	1.56
IC50	30.27	105.22	83.81	101.96	58.77

## 3. Results and discussion 

In this study, self or cross-benzoin reaction of benzaldehyde, 3-hydroxybenzaldehyde, 4-hydroxybenzaldehyde, and 3,5-dihydroxy benzaldehyde was carried out by using different methods. Substituted groups are very effective for the synthesis of benzoin when the electron donating groups are present on benzaldehyde; it is difficult to synthesize the benzoin as known in the literature [5,11,14–16,40–42]. For this purpose, MW, US, and reflux methods were investigated to find out the method to synthesize electron donating substituted group contains asymmetric or symmetric benzoin according to known methods. MW and reflux methods gave low yield or no reactions, and HCN gas evolution was observed. Dozens of trials have been made. However, for the most part, hydroxy substituted benzoin compounds could not be difficult to be synthesized. The US method was the best method to synthesize some of the hydroxy substituted benzoin even in low yield. In the case of using different aldehyde compounds, it is possible to form four alternative benzoins (Table 1). After chromatographic purification, compounds **1-7 (** 39-68% yields, respectively) were obtained as a racemic mixture (Figure) [4,2–71].

^1^H NMR spectra of benzoin compounds gave benzylic -CH(OH) at δ 5.8-6.4 (H-2, bs) ppm and ^13^C NMR spectra revealed peaks at δ 195-198 (C = O) for C-1 and δ 75-78 ppm for C-2, which indicates benzoin structures (Figure).

Hydroxy benzil compounds ( **8-14** ) were synthesized from hydroxy benzoins’ oxidation ( **1-7** ) [72–74] with conc. nitric acid, PCC, and Fehling reagent. It was observed that during the oxidation of benzoin compounds, they decomposed to aldehydes or benzil compounds rearranged to benzylic acids. It has been known that substituted benzoin/benzil compounds decompose during the oxidation [12,13,32, 62,67]. Therefore, the yields of benzil reactions were found in the range of 18%–45%. The disappearance of the benzylic proton peak at δ 5.8–6.4 ppm and benzylic carbon peak at δ 75–78 ppm for the hydroxy benzoin compounds, and the appearance of 1,2-dione carbon peaks at δ 192-195 ppm (C = O) for C-1 and C-2 indicated the hydroxy benzil structures [62, 75] (Figure).

The reaction of hydroxy benzoins ( **1-7** ) and benzils ( **8-14** ) with TABG then hydrolysis of acetyl group yielded benzoin-D-glucosides ( **15-20** ) and benzil-D-glucosides ( **22-25** ), respectively [21-22]. Observation of the anomeric proton coupling constant value around *J* = 7-8 Hz in the ^1^H NMR spectrum of the synthesized benzoin/benzil-O- *β* -D-glucosides shows that the D-glucose unit was in the *β* form. ^13^C NMR spectra of benzoin/benzil-O- *β* -D-glucoside compounds indicate anomeric carbon peaks at δ 100–105 ppm, which showed that one or more D-glucose units were attached to benzoin/benzil compounds (Figure). Benzoin-O- *β* -D-glucoside compounds were observed as diastereomeric mixtures, as seen in NMR spectra. Compounds **6** and **18** were observed as an isomeric mixture. In total, 7 benzoin, 7 benzil, and 6/5 benzoin/benzil-D-glucoside compounds were synthesized, respectively. In the synthesis of the D-glucoside derivative of compounds **7** , **13,** and **14** , highly mixed products were obtained and could not be purified. All the synthetic compounds were characterized by NMR (1D and 2D) and ACD NMR program. According to our literature survey, compounds **3** , **5-7** , **10** , **12-13** , and **15-25** have not been found in the literature.

Antioxidant properties of synthesized compounds **1** - **25** were made according to FRAP, CUPRAC, and DPPH methods [49–52], as seen in Table 2. The highest FRAP and CUPRAC values of hydroxy benzoin compounds ( **1-7)** were 2237±58.3 and 1113.33±64 (μg/mL Trolox/gram DW) in compounds **6** and **5** , and the lowest DPPH values for compounds **5** and **4** were found to be 8.12 ± 1.2 mg/mL and 8.16 ± 0.3 mg/mL, respectively. Among the benzil compounds ( **8-14** ), compound **14** (1974 ± 76.9 μg/mL Trolox/gram DW) to FRAP, compounds **8** and **13** (1095.00 ± 18.1 μg/mL Trolox/gram DW) to CUPRAC, and compound **14** (7.38 ± 1.0 mg/mL) to DPPH methods, were found to be the most active compounds. In the benzoin-D-glucoside compounds **(15-20** ), the highest FRAP, CUPRAC, and lowest DPPH values for benzoin-D-glucoside were 2918.75±36.14, 775.58±12.34 (μg/mL Trolox/gram DW), and 7.78±0.12 mg/mL for compound **20** , respectively. When the activities of all compounds according to FRAP, CUPRAC and DPPH were examined, it was seen that compound **20** for FRAP, compound **5** for CUPRAC, and compound **14** for DPPH were the most effective antioxidant compounds. The antioxidant activities for the benzil-O- *β* -D-glucoside ( **21-25** ) showed that compound **25** was the most effective for FRAP (2570.42 ± 25.01 μg/mL Trolox/gram DW) and the compound **23** was the most active to CUPRAC (273.67 ± 27.23, μg.mL^–1^ Trolox/gram DW) and DPPH (17.74 ± 0.35, mg/mL) methods. When looking at the substitution positions of these compounds, it has been found that they are generally more effective when they are substituted at the 3-position.

The antimicrobial activities of the synthesized compounds **1-14** against eight bacteria and one yeast, and compounds **16-25** against ten bacteria and one yeast were evaluated. After the inhibition diameters were observed in mm (data are not shown), the MIC values (µg/mL) were calculated [53–54] (Table 3). The best MIC values for hydroxy benzoin/benzil compounds **6a+b** , **8** , **10,** and **13** were found within the range of 9–52 µg/mL against bacteria *Y. pseudotuberculosis, P. aeruginosa, S. aureus, S. mutans, E. faecalis,* and *B. cere* us. Compounds **6a+b** , **8** , **10** , **11** , **12,** and **13** showed the best antituberculosis activity with the MIC value in the range of 9–18 µg/mL against *M. smegmatis* . Considering the antifungal activity results, compounds **8** and **10-15** showed closer activity against *C. albicans* with 17-51 µg/mL MIC values. The antibacterial activity results of compounds **15-25** (MIC values, 6-250 µg/mL) showed that compounds ( **15-25** ) were generally the most effective against Gram (+) nonpathogenic spore forming bacteria *P. larvae* and *B. suptilis.* None of the tested compounds **15-25** *g* ave any activity against *Y. pseudotuberculosis* , *P. aeruginosa* , and *S. mutans* . Compound **25** showed MIC values in the range of 8–15 µg/mL against bacteria ( *E. coli, M. smegmatis,* and *P. larvae* ). Among the glycoside compounds, compounds **21** and **22** were only active against nonpathogenic *B. subtilis* and *P. larvae* with 12–27 µg/mL MIC value. Only compound 15 was found to be active against *C. albicans* with 25 µg/mL MIC value among the glycoside compounds. It has been observed that compounds carrying –OH/-H as the -R group in the meta position compared to the carbonyl in their structure and the meta position in the other phenyl ring are more active. 

Acetylcholinesterase [55], butyrylcholine esterase [55], tyrosinase [56], α-amylase [58], α-glucosidase inhibition [58] activity results were made according to spectrophotometric methods. IC_50_ values were calculated and given in Table 4. The result of enzyme inhibition (ACh, BCh, tyrosinase, α-amylase, and α-glucosidase) for hydroxy benzoin/benzil ( **1-14** ) gave that compound **4/10** , **2** , **6a+b /10** , **7/9** , and **1/9** were the most active. Their IC_50 _values were within the range of 25.45–110.96 µg/mL, and 38.90–219.82 µg/mL, respectively. The five different enzyme inhibition (ACh, BCh, tyrosinase, α-amylase, and α-glucosidase) of benzoin/benzil-O- *β* -D-glucosides ( **15-25** ) resulted that compounds **19/23** , **16/25** , **19/21** , **19/23** , and **20/24** were the most active. Their IC_50 _values were within the range of 10.52 –188.93 µg/mL, and 58.90–133.86 µg/mL, respectively. In the enzyme inhibition study, compounds **19** and **2** against ACh and BCh, compounds **7** and **12** against α-amylase and α-glucosidase, and compound **19** against tyrosinase showed activity as much as galantamine, acarbose, and kojic acid standards used, respectively. When these results are compared with the used standards, compounds **19** , **2** , **7** , **12** , and **19** could be used as ACh, BCh, tyrosinase, α-amylase, and α-glucosidase inhibitors, respectively. The highest activities were seen by the compounds bearing the 4-OH, 3-OH substituent on the phenyl rings considering the structure-activity relationship. It is known in the literature that many different hydroxy phenolic compounds show biological activity in a wide spectrum range [1,71].

HeLa test results of compounds **4** , **12** , **18a+b** , and **25** showed that only compound **4** (IC_50_ 42.67 µg/mL) had a strong antiproliferative effect on cancer cells (Table 5). However, the toxicity caused by compound **4** on the normal cell line was examined (IC_50_ 30.27 µg/mL), it was found to be toxic. The inhibitor concentrations (IC_50_) of the compounds **4** , **12** , **18a+b** , and **25** were compared on the HeLa cancer cell, compound **4** (IC_50_ 42.67 µg/mL) had a strong antiproliferative effect as cisplatin (IC_50_ 42.76 µg/mL). The growth inhibition (GI_50_) of compound **4** (GI_50_ 1.12 µg/mL) on the HeLa cancer cell was as the control compound cisplatin (GI_50_ 1.13 µg/mL) [59–61]. The inhibitor concentrations (IC_50_) of the compounds **4** , **12** , **18a+b** , and **25** on the RPE normal cell line gave that compound **4** (IC_50_ 30.27 µg/mL) had better antiproliferative activity than control compound cisplatin (IC_50_ 58.77 µg/mL). Comparison of growth inhibition (GI_50_) on the RPE cancer cells, compound **4** (GI_50_ 1.15 µg/mL) showed growth inhibition as much as cisplatin (GI_50_ 1.56 µg/mL).

## 4. Conclusion

In this study, benzoin reaction was carried out in a modified ultrasonic bath. Antioxidant activities of all compounds were compared according to FRAP, CUPRAC, and DPPH methods. It was found that compound **21** (2918.75±36.14 μg/mL Trolox/gram DW) for FRAP, compound **5** (1113.33 ± 64.9 μg/mL Trolox/gram DW) for CUPRAC, and compound **15** (7.38±1.0 mg/mL) for DPPH were the most effective antioxidant compounds. Hydroxy benzoin/benzil compounds **1-14** were more effective against test microorganisms in the antimicrobial activity among the synthesized compounds **1-25** . Compound **24** showed the only antitubercular activity with the MIC value of 15 µg/mL against *M. smegmatis,* and compound **15** was found to be active against only *C. albicans* with 25 µg/mL MIC value. Thus, compounds **24** and **15** could be used as the standard for *M. smegmatis* and *C. albicans* , respectively. Enzyme inhibition study showed that compounds **19** and **2** against ACh and BCh, compounds **7** and **12** against α-amylase and α-glucosidase, and compound **19** against tyrosinase gave the activity as much as galantamine, acarbose, and kojic acid standards used, respectively. However, it has been observed that all compounds synthesized show different levels of activity against tested enzymes. HeLa and RPE cancer cell activities of compound **4** were observed as much as cisplatin. Biological activity studies of compounds **1-25** showed benzoin and benzil analogs were more active when the substituents in the *meta* positions. Compounds **3** , **5-7** , **10** , **12-13** , and **15-25** are new, and their biological evaluation was made first time in this work.
